# Updates from Our Institutional Experience with Thyroid Nodules Diagnosed as Metastases

**DOI:** 10.3390/diagnostics13142388

**Published:** 2023-07-17

**Authors:** Esther Diana Rossi, Carmine Bruno, Pietro Tralongo, Federica Policardo, Federica Vegni, Angela Feraco, Qianqian Zhang, Alfredo Pontecorvi, Guido Fadda, Celestino Pio Lombardi, Marco Raffaelli, Antonino Mulè, Luigi Maria Larocca

**Affiliations:** 1 Division of Anatomic Pathology and Histology, Università Cattolica del Sacro Cuore, “Agostino Gemelli” School of Medicine, 00168 Rome, Italy; pietro.tralongo@gmail.com (P.T.);; 2 Division of Endocrinology, Università Cattolica del Sacro Cuore, “Agostino Gemelli” School of Medicine, 00168 Rome, Italy; 3 Division of Endocrine Surgery, Università Cattolica del Sacro Cuore, “Agostino Gemelli” School of Medicine, 00168 Rome, Italy

**Keywords:** fine needle aspiration, liquid based cytology, metastatic thyroid carcinoma, immunocytochemistry

## Abstract

Background: Thyroid metastases (TMs) are a rare entity, ranging between 0 and 24% in the autopsy series. In the assessment of the best management, the discrimination between a primary and a metastatic thyroid lesion is crucial. In this regard, fine needle aspiration cytology (FNAC) is likely to play a crucial role especially when ancillary techniques (i.e., immunocytochemistry (ICC) and molecular testing) are carried out. Methods: We searched for all the TMs diagnosed using FNAC and analyzed between 2014 and 2023. The cases were processed with liquid-based (LBC) and ICC and molecular testing performed on LBC-stored material. Results: We reported 2.2% (19 cases) of TMs out of 1022 malignancies. TMs included: 1 larynx carcinoma (LX-Ca), 1 melanoma, 2 breast carcinomas (B-Ca), 3 lung carcinomas (LG-Ca), 4 gastro-intestinal carcinomas (GI-Ca), and 8 clear cell renal carcinomas (CCRC). All patients had a previous cancer history, between 300 and 2 months from the primary cancers. The morphological features were supported by ICC, which were contributive in 100% of cases. All TMs cases were characterized by multiple thyroid nodules except the melanoma case. Four cases underwent total thyroidectomy (1 B, 1 LX, 1 melanoma, and 1 CCRC) whilst 15 TMs were treated with radio-chemotherapy. Conclusions: FNAC empowered the diagnostic workup of patients with TMs avoiding useless surgery. The low sensitivity of cytology might be reinforced by the application of ancillary techniques. We found a predominant rate of kidney metastatic carcinomas, followed by lung and breast. TMs are frequently multifocal and in a context of a systemic disease so a tailored therapy seems to be the best treatment.

## 1. Introduction

In these last decades, fine needle aspiration cytology (FNAC) has clearly shown its crucial diagnostic role in the evaluation of thyroid nodules [1,2,3,4,5]. In fact, together with the ultrasound (US) imaging and further selection of nodules, based on their US patterns, FNAC has been recognized as an important diagnostic tool for classifying thyroid lesions, either benign or malignant entities. Its worldwide application is mostly attributed with the evidence that it is simple, safe and cost-effective, contributing to the correct clinical approach in significantly more than 90% of cases [6]. Furthermore, it has a high sensitivity and negative predictive value as well as a low rate of complications.

In the spectrum of cytological diagnoses, the majority of thyroid nodules are diagnosed as primary thyroid entities using FNAC even though a possible metastatic localization to the thyroid gland should be always kept in mind especially when the morphological features are not univocally in favor of a primary thyroid entity. Not only should the lesions be associated to a previous or current history of malignancy, but they are likely to represent the first evidence of the disease [7,8,9,10]. This latter possibility increases the difficulties in the cytological identification of a thyroid metastatic disease.

Some papers, especially those related to autopsy series, documented that the estimated incidence of thyroid metastatic lesions ranges between 0 and 24%. Furthermore, as emerged from a multi-institutional study of 62 secondary neoplasms to the thyroid gland, the most frequent metastatic diseases seem to be colon, kidney, breast, and lung cancers [9,10,11,12,13,14,15,16].

Despite the fact that thyroid metastases (TMs) may represent an unexpected finding, either cytological or histological series have assessed that TMs account for 1.4% to 2.5% of all thyroid cancers [16,17,18,19,20,21]. However, some of the drawbacks and pitfalls in the cytological interpretation of TMs can be overcome by a combined evaluation of the clinical history, ultrasound evaluation and cytological interpretation. To note, the possible application of ancillary techniques (i.e., immunocytochemistry (ICC) and molecular analysis) significantly empowers the diagnostic role of FNAC [21,22,23,24,25,26,27,28]. In fact, a wise use of ancillary techniques, especially ICC, is likely to support the morphological hypotheses ending up with an increased diagnostic accuracy and sensitivity.

In 2014, our group published the results of a TMs series, diagnosed in the period between 2000 and 2013 [3]. The comparative evaluation of our yields with other series documented a different trend in the histological patterns and diagnoses of the TMs. In fact, whilst several reported series underlined the predominant rate of renal cell carcinomas (RCC), our yields assessed that gastro-intestinal as well as lung and breast cancers were the most common TMs, with a minority of RCC. Based on those yields, we decided to retrospectively evaluate the results of TMs related to the period between 2014 and 2023 and to compare them with the previous 13 years of experience.

## 2. Material and Methods

We conducted a retrospective, computerized search of all metastatic thyroid cytological cases recorded in the files of the Division of Anatomic Pathology and Histology of the Catholic University, “Agostino Gemelli” Hospital of Rome (Italy) between January 2014 and February 2023. A global cohort of 19 (1.8%) cases out of 1022 total thyroid malignancies were diagnosed as positive for malignancy (PM), favoring a metastatic localization, using liquid-based cytology (LBC). We searched for cases with a known history of malignancies or primary suggested diagnoses of TMs with FNAC. In our institution, all the nodules were evaluated under sonographic guidance (US) by pathologists, surgeons, and endocrinologists. All the cases were processed with the LBC method Thin Prep 2000^TM^ (Hologic Co., Marlborough, MA, USA). Each FNAC was performed with two passes for each lesion, using 25 to 27 G needles without any rapid adequacy assessment of the material.

The size of nodules ranged from 0.7 cm to 7.0 cm and they were all found during routine US thyroid check-ups performed in the Centre for Thyroid Diseases of the Departments of Endocrinology and Endocrine Surgery of the Catholic University. Regarding the use of the LBC method, all the patients had been appropriately informed for processing their aspiration samples and a written informed consent was signed by each of them. Our study followed the tenets of the Declaration of Helsinki and we received the internal ethics approval for the study.

Concerning the LBC method, the aspirated material was processed according to the manufacturer’s suggestions and following the standard method that we have been using since the introduction of LBC in our institution in 2000. The resulting slide was fixed in 95% ethanol and stained with Papanicolaou while the remaining material was stored in the PreservCyt^TM^ solution at room temperature for 3 to 4 months to be used for eventual additional investigations or preparation of additional slides. These ancillary techniques can be performed when the remaining material is at about 2 mL eluted in 5 mL of PreservCyt solution. The adequacy was defined, according to the British RCPath classification, by six groups of epithelial cells within the submitted slides, each of them with at least 10 well-visualized epithelial cells [17].

At the beginning, the cytological cases were classified according to the Italian Working Group SIAPEC-IAP classification and then reclassified for scientific purposes, according to the Thyroid Bethesda System. Albeit the differences in classification systems, the selection of metastatic lesions did not incur any discrepancies, and they all belonged to the malignant category regardless of the different classification systems [17,18].

Patients with lymphomas and malignant lesions that invade the thyroid to contiguity were excluded from our series. We excluded any thyroid lymphoma because, as for the previous paper, we wanted to compare the epithelial metastases. Furthermore, we did not include any thyroid infiltration from a contiguous malignancy because we wanted to analyze the thyroid metastatic lesions mimicking a thyroid primary neoplasm instead of analyzing an infiltrative process to the thyroid. In our institution, all the cytological and histological sections were reviewed by two expert cytopathologists and those cases whose interpretation was equivocal were submitted to diagnostic judgment until a final agreement was achieved. In this specific series, there was not any disagreement that needed to be re-evaluated. The follow-up included a period between 2 and 120 months.

### 2.1. Immunocytochemistry

Immunocytochemical stainings were carried out with the avidin-biotin peroxidase complex using a selection of specific antibodies mostly based on the morphological diagnostic hypotheses. The immunocytochemical method was standardized according to the manufacturer’s protocol adopted in our institution and previously published [3,22]. Positivity was assessed for each cytological case when at least a few tumoral cells (5%) showed a strong cytoplasm or nuclear positivity based on the specific immunomarker used. This cut-off percentage was chosen in order to avoid false positive results and lined with the reported stained data on the histological diagnoses. Positive controls and negative controls were selectively used in agreement with each specific immunomarker. For each case, a selection of immunomarkers has been applied. We did not report a standard number of immunomarkers used for each case both on LBC but they ranged from two to eight including all the possible differential diagnoses.

### 2.2. Histology

The surgical specimens were fixed in 10% buffered formaldehyde, embedded in paraffin and the 5 micron-thick sections were stained with haematoxylin-eosin. The concordance of immunohistochemistry between the primary surgical carcinomas and TM cytological samples was 100%. All the fibro-adipose tissue close to the thyroid gland was included for lymph node research and evaluation.

### 2.3. Statistical Analysis

Statistical analysis was performed by using a commercially available statistical software package (GraphPad Prism 8, La Jolla, CA, USA) for iOS (Apple, Cupertino, CA, USA). The comparison of categorical variables was performed by Fisher’s exact test when appropriate. A *p* value less than 0.05 were considered significant.

## 3. Results

**Figure 1:**
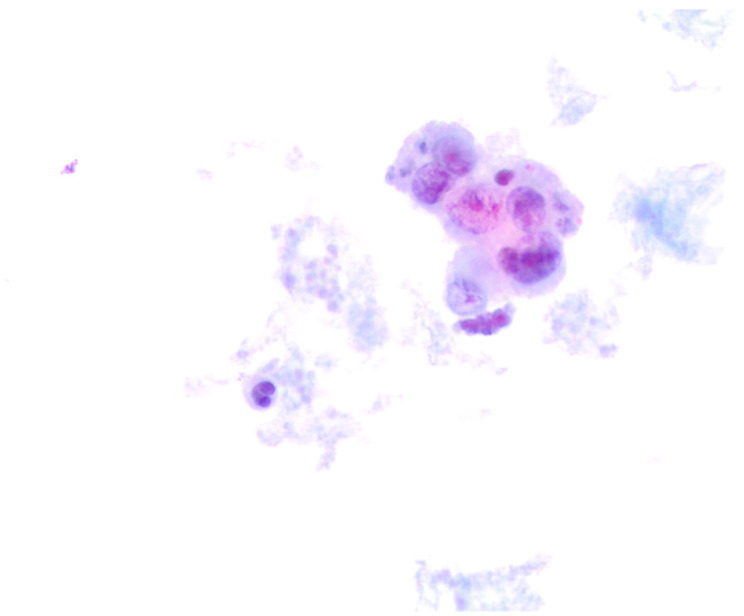
Cytological details from a metastatic melanoma. The cases was hypocellular with a few large pleomorphic cells, characterized by large and eccentrically located nuclei (400× liquid-based cytology, H & E).

**Figure 2:**
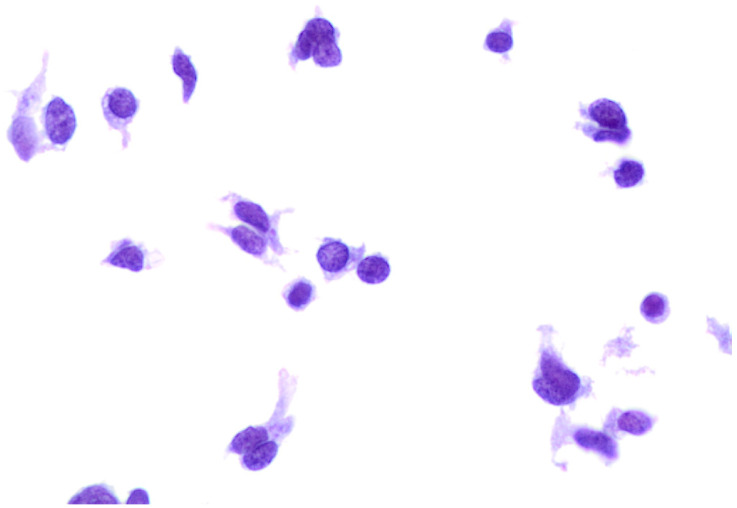
Cytological details from a metastatic small cell lung carcinoma. The cases was hypercellular with isolated and small clusters of malignant cells, characterized by nuclear molding, dark chromatin, and scant cytoplasm (400× liquid-based cytology, H & E).

During the reference period, between January 2014 and February 2023, we found 19 metastatic thyroid FNACs out of 30,006 thyroid FNACs (0.06%). Those metastatic lesions represented 1.8% of 1022 thyroid malignant diagnoses. The series comprised 13 (68.4%) female and 6 (31.6%) male patients with a median age of 65 years old (Table 1). No significant difference was found in the comparative analysis for age and gender. The lesions were clinical and radiological detected and the majority of them were in the context of multiple thyroid nodules. To note, one case was characterized by a single nodule, then diagnosed as a metastatic melanoma. All the 19 cases were diagnosed as “positive for malignancy-favoring metastatic localization”. In this series, we found: 1 metastatic laryngeal cancer, 1 melanoma (Figure 1), 2 breast carcinomas, 3 lung cancers including 2 adenocarcinoma and 1 small cell carcinoma (Figure 2), 4 gastro-intestinal adenocarcinomas (mostly colon adenocarcinoma) and 8 metastatic clear cell renal carcinomas (CCRC). All the patients had a previous histological diagnosis of a primary carcinoma. We did not refer any metastatic lesions from unknown primary carcinomas. Table 1 shows the distribution of the diagnosis for each primary carcinoma and the detailed distribution of the primary malignancies. Specifically, all of the renal cell metastases were associated with a localization of a clear cell renal carcinoma; the four gastro-intestinal carcinomas included four colon adenocarcinomas. In the group of lung carcinomas, we had two cases of lung adenocarcinomas (including one high-grade adenocarcinoma) and one small cell carcinoma (SmCC). Our two breast carcinomas were diagnosed as G3 ductal histotype carcinomas. The larynx carcinoma was diagnosed as squamous poorly differentiated carcinoma (Table 1). Only 4 out of 19 patients (21.5%) underwent a total thyroidectomy.

In Table 2, we analyzed the clinical–pathological features of the TMs, including also the immunomarkers carried out on LBC-stored material. According to the clinical history, the mean time elapsed from the primary tumor diagnosis to the detection of thyroid metastasis was 49 months with only 3 out of 19 cases with a synchronous detection (2 months). The overall survival time reported that 14 patients are still alive with the shortest survival time (11 months) in a patient with a lung adenocarcinoma. The patient with the diagnosis of metastatic melanoma is still alive, also because the diagnosis was a recent discovery of both primary and metastatic localization.

In Table 3, we reported the immunomarkers based on the clinical history of each patient and the expression of them in the different entities. To note, the adoption of an immunopanel of markers was performed in all the cases.

**Figure 3:**
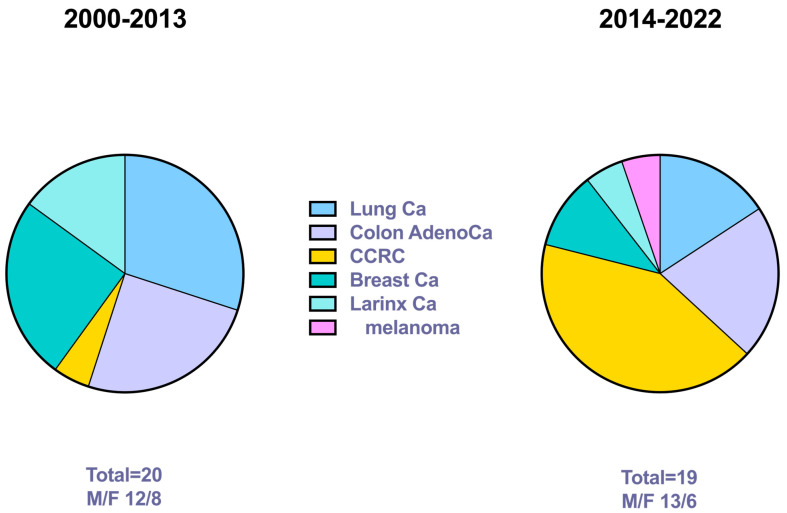
The figure shows the distribution of thyroid metastases in the two reference periods with the statistical *p* values.

The conclusive diagnosis resulted from a combination of morphological features, IIC yields, and clinical history in all our 19 cases. Notably, none of the cases was positive for Thyroglobulin (Table 3), confirming that in the case of a negative Thyroglobulin stain, a detailed evaluation of other immunomarkers is mandatory. In Table 4 we compared the figures from the previously published series with the current yields (Figure 3). The results showed that the number of TMs was stable per decade. Nonetheless, we found some consistent differences with a significant *p* for the CCRC. In fact, the current series is characterized by a high number of CCRCs, followed by colon adenocarcinoma. Notably, the gap between the primary RCC and the metastatic localization ranged from 20 to 30 years.

## 4. Discussion

The main purpose of the current paper is to evaluate our institutional experience in the diagnosis of TMs, with reference to the period between 2014 and 2023. Furthermore, we correlate the yields with our previous results referring to a published series of TMs in the period between 2000 and 2013 [3].

Unexpectedly, in the first series, we found a predominant number of gastrointestinal metastatic adenocarcinomas and only one clear cell renal carcinoma. Based on those data, we decided to compare them with our further cases from 2014 to 2023 and to discuss similarities and/or differences between the two series.

Data from the autopsy series have documented that TMs range between 0 and 24% of thyroid nodules, whilst according to the clinical series, mostly published by Cheung et al. and Montero et al. [7,8], the reported incidence varies between 0.1 to 3%.

According to our results, we found 2.2% TMs in the first series versus 1.8% in the second reference period. Apart from slight differences, these two figures showed an overlap in the numbers of TMs, with quite stable figures in the different decades. In fact, these percentages, belonging to two consecutive decades, have confirmed the trend that the thyroid gland represents a rare metastatic site for many different primary carcinomas despite its rich vascular structure and supply [8,28,29,30,31]. This unusual low percentage of metastases might be attributed to different factors including the high iodine environment and hyperoxic status as well as some mechanical difficulty from the tumor cells to reach the thyroid parenchyma [8]. Furthermore, in the current series we confirm a stronger evidence of a metachronic metastatic process with only three patients who developed thyroid metastases in a period shorter than 2 months [8,11]. An important challenge is the evaluation of the role of FNAC in the diagnosis of TMs. Whilst the majority of published papers have assessed the role of FNAC in the diagnostic evaluation of primary thyroid lesions, few papers have documented its role in the interpretation of a metastatic localization to the thyroid. This limitation is likely to be ascribed to the fact that primary thyroid lesions represent the prevalent percentage of all thyroid diagnoses, with only a minority of them reported as metastases. As a consequence, a metastatic localization might be misdiagnosed, especially when a complete clinical history is not provided and/or unknown. Furthermore, both the morphological and ultrasound findings do not show any consistent difference between primary and metastatic lesions, including those cases with a multifocal/multinodular pattern [10,11,12,13].

Few papers have discussed the role of FNAC in the diagnosis of TM, mostly suggesting that the crucial step is represented by the ability to obtain the adequate amount of diagnostic neoplastic cells [7,9]. Both our series have shown a very high diagnostic accuracy, mostly attributed to the number of passes per each lesion (never less than two passes) but also because TMs are frequently highly cellular solid lesions, reducing dramatically the rate of non-diagnostic cases. In our series, the non-diagnostic rate was 0%, confirming that an adequate sample is crucial for the diagnostic interpretation. To note, our data also highlighted the reliability and feasibility of LBC as an alternative and valid diagnostic cytologic method.

In a series by Wysocka et al., the authors found TMs and nodal metastases (NM) in 57 patients (0.3% of all examined; 40 TM, 22 NM, 5 both). The authors assessed that their frequency was higher in the group of patients with a known history of primary cancer compared with the group of unknown primary lesions (2.9% vs. 0.1% in C–, *p* < 0.0001) [23].

A revision of literature that referred to the years before 2014, reported a medium number of TMs ranging from 14 and 22 cases over a period between 55 years and 7 years [7,20,21]. Both our series, each of them including 20 cases and 19 cases, respectively, are in line with these yields. Specifically, both our series were composed of cases with a previous diagnosis of malignancy, for which FNAC has been crucial for a correct diagnosis and for the best tailored and personalized management. Specifically, all of them had a conclusive diagnosis, concurring with the specific management of each patient.

Despite the fact that different papers highlighted an equal gender distribution of TMs, we have to underline a slightly higher incidence in the female population. In agreement with the majority of the series, we have also documented a presentation in the sixth or seventh decade of life. Nonetheless, concerning the TMs from the current series, we found some significant discrepancies with the results obtained in the first period. In detail, the current series supported the evidence that CCRC is the most frequent thyroid metastasis (42.1%) representing a cause of common pitfalls. In a previous multicenter study, including 7 United States and European medical centers, also involving our institution, 3 out of 9 CCRC cases were misdiagnosed as follicular neoplasms [1]. The failure is due to different concurrent causes including the lack of the previous clinical history about a CCRC and the evidence of a single nodule in the thyroid, which is more likely in favor of a primary thyroid lesion. In fact, the interval between a primary CCRC and its metastasis could be very long, with a median time of 20–30 years. For that reason, the knowledge of the clinical history is crucial for a correct diagnosis, which is made by the combination of morphological interpretation and the support of the ICC on the cytological material. In our eight cases, the interval between the primary CCRC and metastatic localization was >25 years. Furthermore, in two of our renal TM cases, we found an intra-adenomatous localization of the metastatic CCRC, as also reported in other case reports. Notably, also in the current series, we found a significant (four cases) number of gastro-intestinal metastases, mostly colon adenocarcinomas, even though we did not record the same preponderant evidence of gastro-intestinal carcinoma metastasizing to the thyroid gland comparing with the first series [3]. Considering the entire range of 2 decades, including the period from 2000 to 2023, we have diagnosed 9 gastro-intestinal TM cases, representing, to the best of our knowledge, the largest series of gastro-intestinal TMs reported in literature.

Hence, in our series, as also documented in other publications, only one case diagnosed as a metastatic melanoma was defined by a single solitary nodule, characterized by single-patterned and pleomorphic cells, which might lead to several differential diagnoses, including anaplastic thyroid carcinoma. Despite the fact that 100% of our cases were diagnosed as malignant-favoring a metastatic localization, the diagnosis of the primary carcinoma/adenocarcinoma cannot be further classified by the morphological evaluation alone. Regardless of the previous clinical history, the support of ancillary techniques, especially ICC, performed on LBC-stored material, is useful for making the definitive diagnosis [21,22,23,24,25,26,27,28]. As documented by several previous publications, we have a long-standing experience with ICC on LBC, confirming its valuable diagnostic role in the TM series [3,22]. In fact, the possibility to have LBC-stored material helps in carrying out many different immunomarkers, with the purpose to include and/or exclude several differential diagnoses. It is important to underline that ICC should always be interpreted and combined with the morphological features. In absence of a primary clinical history, the evidence of morphological features that are not in favor of a primary thyroid tumor should be combined with the ICC analysis. In those cases, characterized by the lack of TTF-1 and Thyroglobulin, a larger number of immunomarkers including keratins, mesenchymal, lymphoid and melanocytic markers has to be evaluated.

The last point is dedicated to the management of thyroid metastases, which is, however, not univocally defined by specific surgical guidelines. Whilst some papers suggest a lobectomy in cases of solitary thyroid metastasis, others propose total thyroidectomy in a multifocal metastatic process [31,32]. Notwithstanding the lack of a consensus, in our experience, thyroidectomies are likely to reduce a further dissemination of the metastatic process but they are not helpful in terms of long-term patients’ survival [9]. In the current series, we included 4 out of 19 thyroidectomies performed because of the mechanical/obstructive symptoms and the absence of any further metastatic localization followed by a radio and/or a chemotherapy approach.

In conclusion, for those cases with a primary known cancer, FNAC shows high accuracy in the diagnosis of TM, especially when it is combined with the application of ancillary techniques which may facilitate to tailor the best and appropriate management (including chemotherapy and radiotherapy) for a metastatic patient [33,34,35,36]. The current series has confirmed that CCRC, followed by gastro-intestinal carcinomas, are the most frequent primary lesion. An adequate sampling is mandatory for a conclusive diagnosis and it should be always combined with the clinical history and the ultrasound pattern. For those cases without a primary story of malignancy, the lack of morphological features in favor of a primary thyroid lesion, should be followed by the performance of ICC for TTF1 and Thyroglobulin, whose negativity will lead to an ampler set of immunomarkers.

## Figures and Tables

**Table 1:** Summary of clinical–pathologic data.

Clinical–Pathological Feature	Proportion (*n* = 19 Cases)
Age	
Mean	55.4 years
Median	55.5 years
Range	16–82 years
Gender	
Male	6 (31.5%)
Female	13 (68.5%)
Cytology diagnosis	
Malignant-Favoring metastasis	19 (100%)
Histopathology	
Malignant	19 (100%)
Lung Carcinoma	3
Breast Carcinoma	2
Renal Cell Carcinoma	8
Colon Adenocarcinoma	4
Larinx Carcinoma	1
Melanoma	1

**Table 2:** Clinical and morphological data of our 19 thyroid metastatic lesions.

Case	Gender	Age	Primary Cancer	Thyroid Cytology of PM-Favoring TM	Time of Thyroid Mets (Month)	Survival (Month)	Thyroid Surgery
1	M	56	Colon Ca	Met Squamous Ca	6	26	No
2	F	22	Colon Ca	Met Squamous High-Grade Ca	5	30	no
3	M	44	Colon Ca	Met Adenoca	8	27	No
4	M	71	Colon Ca	Met Adenoca	36	36–alive	No
5	F	89	Lung Adenoca	Met High-Grade Adenoca	4	14	No
6	F	67	Lung Ca	Met Adenoca	7	10	no
7	M	58	Lung Ca	Met Small Cell Carcinoma	24	72–alive	no
8	F	57	Breast Ca	Met Ductal Ca	60	42	yes
9	F	60	Breast Ca	Met Ductal Ca	100	120	yes
10	M	74	Larinx Ca	Met Squamous Ca	12	72–alive	yes
11	M	66	Melanoma	Met Melanoma	2	5–alive	No
12	F	65	Clear Cell Renal Ca-CCRC	Met CCRC	58	96–alive	yes
13	F	72	Clear Cell Renal Ca-CCRC	Met CCRC	94	102–alive	no
14	F	59	Clear Cell Renal Ca-CCRC	Met CCRC	90	97–alive	no
15	F	77	Clear Cell Renal Ca-CCRC	Met CCRC	102	7110	no
16	F	69	Clear Cell Renal Ca-CCRC	Met CCRC	110	115	no
17	F	67	Clear Cell Renal Ca-CCRC	Met CCRC	88	99–alive	no
18	F	75	Clear Cell Renal Ca-CCRC	Met CCRC	76	103	no
19	F	71	Clear Cell Renal Ca-CCRC	Met CCRC	80	101	no

Legend: Ca: carcinoma; Adenoca: adenocarcinoma; PM-favoring TM: positive for malignancy- favoring thyroid metastasis; Met: metastatic; CCRC: clear cell renal carcinoma.

**Table 3:** Correlation between the primary tumors and the cytological metastases morphological and immunocytochemical profiles.

Primitive Cancers	No. of Cases	Clinical Thyroid Evidence	FNAC (PM-Favoring TM)	Antibody Tested on LBC
Colon Adenocarcinoma	4	Nodule	Met Adenoca	Positive: CAM 5.2; CK20; CDX2 Negative: Thyroglob; TTF-1
Breast Ca	2	Nodule	Met Ductal Adenoca	Positive: CK7; GCDFP15; ER; PR; E-Cadherin Negative: Thyroglobulin; TTF-1
Lung Ca	3	Nodule	Met Adenoca and SmCC	Positive: TTF-1; CAM5.2; CK7; CDX2 Negative: Thyroglobulin; S100 Positive: TTF1; CAM5.2; Chromogranin; Synaptophysin; CK7 Negative: Thyroglobulin; S100
Larinx	1	Nodule	Met Squamous Ca	Positive: EMA; CAM 5.2; AE1/AE3 Negative: Thyroglobulin; TTF-1; Calcitonin; HBME-1; Galectin3
Renal Ca	8	Nodule	Met Renal Clear Cell Carcinoma	Positive: EMA; CAM 5.2; Vimentin Negative: Thyroglobulin; TTF-1
Melanoma	1	Nodule	Met Melanoma	Positive: S100; MelanA; HMB45 Negative: Thyroglobulin; TTF1

None of these nodules was a single thyroid nodule. Legend: Ca: carcinoma; Adenoca: adenocarcinoma; LBC: liquid-based cytology; PM-favoring TM: positive for malignancy-favoring thyroid metastasis; Met: metastatic.

**Table 4:** Comparison between the two series from the consecutive periods. The first series had already been published.

	Series 2000–2013	Series 2014–2022	*p*
No. of cases	20	19	/
F/M	12/8	13/6	*p* = 0.74
No. of Lung Ca Colon Adenoca CCRC Breast Ca Larinx Ca Melanoma	6 (30%) 5 (25%) 1 (5%) 5 (25%) 3 (15%) 0 (0%)	3 (15.8%) 4 (21.1%) 8 (42.1%) 2 (10.5%) 1 (5.2%) 1 (5.2%)	*p* = 0.45 *p* = 0.99 *p* = 0.008 *p* = 0.41 *p* = 0.60 *p* = 0.49
No. of thyroidectomy	2	4	/
ICC	20	19	/

Legend: F/M: female/male; Ca: carcinoma; Adenoca: adenocarcinoma; CCRC: clear cell renal carcinoma; ICC: immunocytochemistry.

## Data Availability

Not applicable.

## References

[1] Pusztaszeri M., Wang H., Cibas E.S., Powers C.N., Bongiovanni M., Ali S., Khurana K.K., Michaels P.J., Faquin W.C. (2015). Fine needle aspiration biopsy of secondary neoplasms of the thyroid gland: A multi-institutional study of 62 cases. Cancer Cytopathol..

[2] Ravetto C., Colombo L., Dottorini M.E. (2000). Usefulness of fine- needle aspiration in the diagnosis of thyroid carcinomas. A retro-spective study in 37,895 patients. Cancer Cytopathol..

[3] Rossi E.D., Martini M., Straccia P., Gerhard R., Evangelista A., Pontecorvi A., Fadda G., Maria Larocca L., Schmitt F. (2015). Is thyroid gland only a “land” for primary malignancies? Role of morphology and immunocytochemistry. Diagn. Cytopathol..

[4] Nardi F., Basolo F., Crescenzi A., Fadda G., Frasoldati A., Orlandi F., Palombini L., Papini E., Zini M., Pontecorvi A. (2014). Italian consensus for the classification and reporting of thyroid cytology. J. Endocrinol. Investig..

[5] Ali S.Z., Cibas E.S. (2010). The Bethesda System for Reporting Thyroid Cytopathology.

[6] Bellantone R., Lombardi C.P., Boscherini M., Alesina P., Rosato L. (2000). Metastasi della tiroide ed alla tiroide. La Patologia Chirurgica della Tiroide e delle Paratiroidi.

[7] Kim T.Y., Kim W.B., Gong G., Hong S.J., Shong Y.K. (2005). Metastasis to the thyroid diagnosed by fine-needle aspiration biopsy. Clin. Endocrinol..

[8] Fujita T., Ogasawara Y., Doihara H., Shimizu N. (2004). Rectal adenocarcinoma metastatic to the thyroid gland. Int. J. Clin. Oncol..

[9] Papi G., Fadda G., Corsello S.M., Corrado S., Rossi E., Radighieri E., Miraglia A., Carani C., Pontecorvi A. (2007). Metastases to the thyroid gland: Prevalence, clinicopathological aspects and prognosis: A 10-year experience. Clin. Endocrinol..

[10] Mirallié E., Rigaud J., Mathonnet M., Gibelin H., Regenet N., Hamy A., Bretagnol F., de Calan L., Le Néel J.-C., Kraimps J.-L. (2005). Management and prognosis of metastases to the thyroid gland. J. Am. Coll. Surg..

[11] De Ridder M., Sermeus A.B., Urbain D., Storme G.A. (2003). Metastases to the thyroid gland—A report of six cases. Eur. J. Intern. Med..

[12] Wood K., Vini L., Harmer C. (2004). Metastases to the thyroid gland: The Royal Marsden experience. Eur. J. Cancer Surg..

[13] Cichoń S., Anielski R., Konturek A., Barczyński M., Cichoń W. (2006). Metastases to the thyroid gland: Seventeen cases operated on in a single clinical center. Langenbeck’s Arch. Surg..

[14] Haraguchi S., Hioki M., Yamashita K., Orii K., Matsumoto K., Shimizu K. (2004). Metastasis to the thyroid from lung adenocarcinoma mimicking thyroid carcinoma. Jpn. J. Thorac. Cardiovasc. Surg..

[15] Delitala A.P., Vidili G., Manca A., Dial U., Delitala G., Fanciulli G. (2014). A case of thyroid metastasis from pancreatic cancer: Case report and literature review. BMC Endocr. Disord..

[16] Calzolari F., Sartori P.V., Talarico C., Parmeggiani D., Beretta E., Pezzullo L., Bovo G., Sperlongano P., Monacelli M., Lucchini R. (2008). Surgical treatment of intrathyroid metastases: Preliminary results of a multicentric study. Anticancer Res..

[17] Ali S., Cibas E.S. (2018). The Bethesda System for Reporting Thyroid Cytopathology.

[18] Fadda G., Basolo F., Bondi A., Bussolati G., Crescenzi A., Nappi O., Nardi F., Papotti M., Taddei G., Palombini L. (2010). Cytological classification of thyroid nodules. Proposal of the SIAPEC-IAP Italian Consensus Working Group. Pathologica.

[19] Wychuis A.R., Beahrs O.H., Woolner L.B. (1964). Metastasis of Carcinoma to the Thyroid Gland. Ann. Surg..

[20] Nakhjavani M.K., Gharib H., Goellner J.R., van Heerden J.A. (1997). Metastasis to the thyroid gland. A report of 43 cases. Cancer.

[21] Smith S.A., Gharib H., Goellner J.R. (1987). Fine needle aspiration: Usefulness for diagnosis and management of metastatic carcinoma to the thyroid. Arch. Intern. Med..

[22] Fadda G., Rossi E.D., Mulè A., Miraglia A., Vecchio F.M., Capelli A. (2006). Diagnostic Efficacy of Immunocytochemistry on Fine Needle Aspiration Biopsies Processed by Thin-Layer Cytology. Acta Cytol..

[23] Wysocka K., Małyska A., Zadworny D., Sporny S., Klencki M., Woźniak-Oseła E., Koptas W., Popowicz B., Słowińska-Klencka D. (2015). Secondary tumours revealed during fine needle aspiration of the thyroid—Analysis of prevalence and characteristics of ultrasound image. Endokrynol. Pol..

[24] Leung S.W., Bédard Y.C. (1996). Immunocytochemical staining on ThinPrep processed smears. Mod. Pathol..

[25] Dabbs D.J., Abendroth C.S., Grenko R.T., Wang X., Radcliffe G.E. (1997). Immunocytochemistry on the Thin Prep processor. Diagn. Cytopathol..

[26] Tabbara S.O., Sidawy M.K., Frost A.R., Brosky K.R., Coles V., Hecht S., Radcliffe G., Sherman M.E. (1998). The stability of estrogen and progesterone receptor expression on breast carcinoma cells stored as PreservCyt suspensions and as ThinPrep slides. Cancer Cytopathol..

[27] Nonaka D., Tang Y., Chiriboga L., Rivera M., Ghossein R. (2008). Diagnostic utility of thyroid transcription factors Pax8 and TTF-2 (FoxE1) in thyroid epithelial neoplasms. Mod. Pathol..

[28] Rivera M., Shang C., Gerhard R., Ghossein R., Lin O. (2010). Anaplastic thyroid carcinoma. Morphological findings and PAX-8 expression in cytology specimens. Acta Cytol..

[29] Iesalnieks I., Winter H., Bareck E., Sotiropoulos G.C., Goretzki P.E., Klinkhammer-Schalke M., Bröckner S., Trupka A., Anthuber M., Rupprecht H. (2008). Thyroid Metastases of Renal Cell Carcinoma: Clinical Course in 45 Patients Undergoing Surgery. Assessment of Factors Affecting Patients’ Survival. Thyroid.

[30] Shimaoka K., Sokal J.E., Pickren J.W. (1961). Metastatic neoplasms in the thyroid gland.Pathological and clinical findings. Cancer.

[31] Rosen I.B., Walfish P.G., Bain J., Bedard Y.C. (1995). Secondary malignancy of the thyroid gland and its management. Ann. Surg. Oncol..

[32] Chen H., Nicol T.L., Udelsman R. (1999). Clinically Significant, Isolated Metastatic Disease to the Thyroid Gland. World J. Surg..

[33] Fadda G., Rossi E.D. (2011). Liquid-Based Cytology in Fine-Needle Aspiration Biopsies of the Thyroid Gland. Acta Cytol..

[34] Ort S., Goldenberg D. (2008). Management of Regional Metastases in Well-Differentiated Thyroid Cancer. Otolaryngol. Clin. N. Am..

[35] Nixon I.J., Whitcher M., Glick J., Palmer F.L., Shaha A.R., Shah J.P., Patel S.G., Ganly I. (2011). Surgical Management of Metastases to the Thyroid Gland. Ann. Surg. Oncol..

[36] Chung A.Y., Tran T.B., Brumund K.T., A Weisman R., Bouvet M. (2011). Metastases to the Thyroid: A Review of the Literature From the Last Decade. Thyroid.

